# Sites of instability in the human *TCF3* (*E2A*) gene adopt G-quadruplex DNA structures *in vitro*

**DOI:** 10.3389/fgene.2015.00177

**Published:** 2015-05-11

**Authors:** Jonathan D. Williams, Sara Fleetwood, Alexandra Berroyer, Nayun Kim, Erik D. Larson

**Affiliations:** ^1^School of Biological Sciences, Illinois State UniversityNormal, IL, USA; ^2^Department of Microbiology and Molecular Genetics, University of Texas Health Science Center at HoustonHouston, TX, USA

**Keywords:** G-quadruplex, G4, t(1;19), *TCF3*, *PBX1*

## Abstract

The formation of highly stable four-stranded DNA, called G-quadruplex (G4), promotes site-specific genome instability. G4 DNA structures fold from repetitive guanine sequences, and increasing experimental evidence connects G4 sequence motifs with specific gene rearrangements. The human transcription factor 3 (*TCF3*) gene (also termed *E2A*) is subject to genetic instability associated with severe disease, most notably a common translocation event t(1;19) associated with acute lymphoblastic leukemia. The sites of instability in *TCF3* are not randomly distributed, but focused to certain sequences. We asked if G4 DNA formation could explain why *TCF3* is prone to recombination and mutagenesis. Here we demonstrate that sequences surrounding the major t(1;19) break site and a region associated with copy number variations both contain G4 sequence motifs. The motifs identified readily adopt G4 DNA structures that are stable enough to interfere with DNA synthesis in physiological salt conditions *in vitro*. When introduced into the yeast genome, *TCF3* G4 motifs promoted gross chromosomal rearrangements in a transcription-dependent manner. Our results provide a molecular rationale for the site-specific instability of human *TCF3,* suggesting that G4 DNA structures contribute to oncogenic DNA breaks and recombination.

## Introduction

Transcription factor 3, also called E2A, is a key regulatory protein that institutes transcriptional programs for proper B and T cell differentiation (Kee et al., 2000; Miyazaki et al., 2014). Given well-established regulatory roles in transcription activation, it is not surprising that disruption of *TCF3* or its gene product is associated with malignant transformation. For instance, a translocation event t(1;19) between the *TCF3* and *PBX1* genes results in the expression of a TCF3–PBX1 chimera, which is commonly found in ALL (Hunger, 1996; Aspland et al., 2001; Pui et al., 2004). Genomic studies of Burkitt’s lymphomas found *TCF3* to be among the most mutated genes (Schmitz et al., 2012) and 70% of patient samples were identified with heterozygous *TCF3* deletions in Sezary syndrome cells, an aggressive T cell lymphoma (Steininger et al., 2011). In addition to pre-B cell cancers, TCF3–PBX1 fusion transcripts have been identified in non-small cell lung cancer (Mo et al., 2013), indicating that the impact of this rearrangement likely extends beyond the immune system. While *TCF3* appears to be a hot spot for DNA breaks, and broadly associated with oncogenesis, the mechanisms responsible for driving this genetic instability are undefined.

The distribution of chromosomal breakpoints involved in the t(1;19) translocation suggest that certain sequences are involved in promoting instability. Previous characterization of t(1:19) breakpoints revealed that most of the recombination junctions (16 of 24) cluster in a 5 bp sequence window in *TCF3* (Wiemels et al., 2002). Those breaks were associated with CpG sequences in *TCF3*, but not *PBX1* (Tsai et al., 2008), and these same recombination sites are surrounded by transposable element repeats, specifically a MER20 transposon (Rodić et al., 2013). The non-random distribution of break sites and proximity to DNA repeat motifs suggest that the recombination events involving *TCF3* are influenced at either the DNA sequence or structural level.

DNA repeats can promote instability because of their ability to adopt structural conformations that interfere with normal DNA transactions (Lobachev et al., 2007; Zhao et al., 2010; van Kregten and Tijsterman, 2014). In particular, guanine repeats readily fold into highly stable four-stranded structures called G4 DNA, which promote mutagenesis and recombination (Brooks et al., 2010; Wu and Brosh, 2010; Bochman et al., 2012; Tarsounas and Tijsterman, 2013; van Kregten and Tijsterman, 2014). G4 DNA structures are stabilized by hydrogen bonding between four guanine bases to create a single “tetrad” of guanines. When tandem guanine repeats are present, stacks of tetrads can form within or among DNA strands to build the four-stranded, or quadruplex, structure. The size, stability, and specific type of G4 structure depends upon the characteristics of the repeat sequence and aqueous conditions (Burge et al., 2006).

G-quadruplex DNA structures have been identified in loci involved in both induced and spontaneous genome instability (Maizels and Gray, 2013). At the guanine-rich immunoglobulin switch regions, programmed recombination requires transcriptional activation, and transcribed switch regions can form loops that are stabilized by RNA/DNA hybrids on one strand and G4 DNA on the other (Duquette et al., 2004). Factors involved in this recombination pathway specifically recognize G4 DNA (Larson et al., 2005). In addition to induced recombination events, G4 DNA structures may lead to instability at other guanine-rich genomic loci. In a few examples, G4 DNA structures fold from sequences flanking the translocation break sites in the *HOX11* gene (Nambiar et al., 2013). Similarly, sequences near the break sites in *BCL2* that lead to t(14;18) translocations form G4 structures *in vitro* (Nambiar et al., 2011). This is probably not a feature of just a few unstable genes because in lymphoid cancers G4 sequence motifs were found at multiple rearrangement sites, including the *TCF3* gene (Katapadi et al., 2012). Experimental systems have also directly connected instability of guanine-rich DNA with the formation of G4 structures. For instance, chromosomal rearrangements at guanine-rich human minisatellite sequences were dependent upon G4 formation (Lopes et al., 2011; Piazza et al., 2012), and addition of G4-stabilizing ligands increased the instability at the G4 repeats, but not for other types of sequence repeats (Piazza et al., 2010). Chromosomal rearrangement assays have also been developed to characterize specific loci and factors involved in G4-mediated genome instability (Piazza et al., 2012; Paeschke et al., 2013; Yadav et al., 2014).

Considering the emerging evidence connecting guanine-rich sequences and G4 DNA structures with genome instability, we asked if the known rearrangements of the *TCF3* gene correlate with G4 structure formation. Here, we applied a comprehensive computational analysis of *TCF3* and the translocation partner *PBX1* and found that G4 sequence motifs reside near the major t(1;19) breakpoints. Using multiple methods, we demonstrate here that those sequences fold into highly stable G4 structures *in vitro* and induced site-specific instability in yeast. We also characterize a second site in *TCF3* for G4 formation that is not involved with the t(1;19) translocation, but is instead associated with CNVs. Our results indicate that the site-specific instability of human *TCF3* is governed at least in part by a capacity to form structures at those sites.

## Results and Discussion

### G4 Sequence Motifs Surround Regions of Instability in *TCF3*

Based on a growing body of evidence implicating G4 DNA structures with site-specific rearrangements, we asked if formation of G4 DNA structures could explain the apparent genetic instability of the *TCF3* gene. The positions of 30 different breakpoints in *TCF3* spanning 4 kb, and 16 breakpoints in *PBX1* spanning 12 kb are documented in the Translocations In Cancer database (TICdb; Novo et al., 2007) and the Leiber database (Tsai et al., 2008). We used these sites to analyze and map break positions in relation to G4 sequence motifs. This was accomplished by applying a web-based server program for G4 structure prediction to overlay G4 sequences with known break site positions.

**FIGURE 1:**
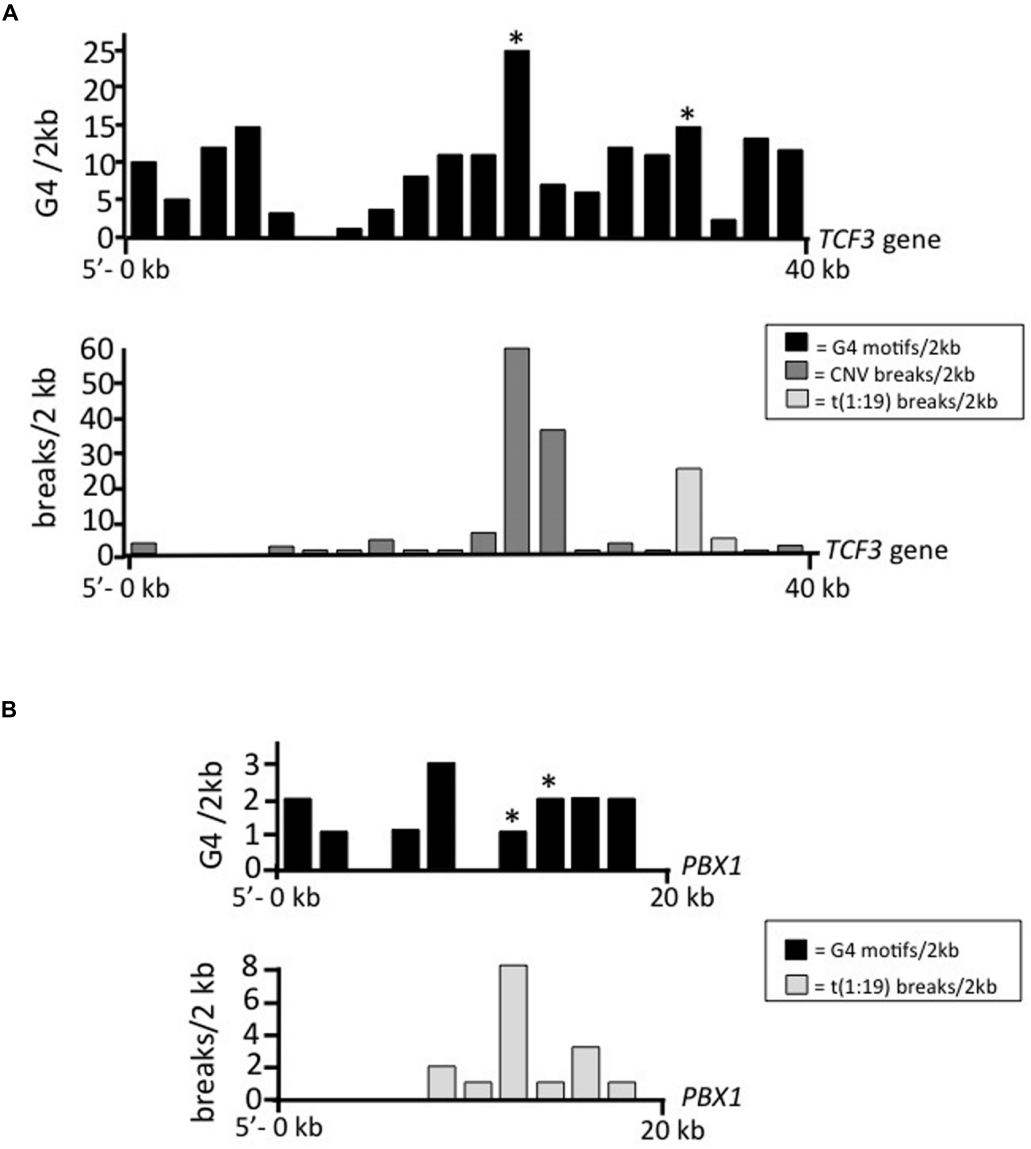
**Genome instability coincides with G4 motifs in *TCF3* and *PBX1*. (A,B)** The number of individual non-overlapping G4 sequence motifs (black) identified in 2 kb windows (Y-axis) graphed according to genetic position (X-axis) within the **(A)** *TCF3* gene or **(B)** 20 kb region of *PBX1* intron associated with the t(1;19) translocation, displayed in the 5′–3′ direction. Regions corresponding to motifs tested for G4 formation are indicated (^∗^). The bar graphs below the *TCF3* and *PBX1* gene diagrams show the relative locations of break sites (light gray) and CNVs (dark gray) identified using 2 kb sequence windows.

Quadruplex forming G-rich sequences (QGRS mapper) is a web-based server program developed to score nucleic acid sequences for G4 forming potential (Kikin et al., 2006). QGRS mapper converts input sequences into scores representing the likelihood for G4 formation, with a maximum score value of 180 given for a run of guanines that is 27 nt long (Kikin et al., 2006). The location of G4 motifs within the queried sequence is an output of the program (Kikin et al., 2006). **Figures 1A,B** diagrams the output for QGRS scoring of *TCF3* and *PBX1* genes. Both strands were analyzed for non-overlapping motifs. Two regions of *TCF3* are associated with genomic instability; the t(1;19) translocation site (which forms the TCF3–PBX1 chimera), and a region rich in breaks connected to CNVs. Both of these regions are intronic. QGRS mapper identified 25 different G4-capable sequences at the CNV site, and 15 G4 sequences surrounding the major t(1;19) break site (**Figure 1A**). An additional region of high guanine repeat density can be found in the 5′ end of the gene, but this site did not correlate with any known breakpoints, suggesting the genetic positioning of G4 sequences influences structure formation, or that instability at that site is not connected with diseases cataloged in existing databases. *PBX1* also contains G4 motifs near the t(1;19) translocation site, but G4 motifs occur at much lower density compared to *TCF3*, with 2 found for *PBX1* (**Figure 1B**) compared to 15 for *TCF3* (**Figure 1A**).

### *TCF3* and *PBX1* G4 Motifs Support G4 Structure Formation *In Vitro*

Considering the breadth of experimental evidence linking site-specific instability with G4 DNA structures (Brooks et al., 2010; Wu and Brosh, 2010; Bochman et al., 2012; Tarsounas and Tijsterman, 2013; van Kregten and Tijsterman, 2014), we asked if the guanine-rich motifs we identified using QGRS mapper also form stable G4 structures in physiological salt and pH conditions. We selected multiple G4 sequence motifs for each break site locus. This includes one repeating G4 motif from a large guanine-rich intron connected with CNVs in *TCF3* (T-lg), three motifs flanking the t(1;19) breakpoint [T-5′, T-3′, and T-3′(2)], and two motifs next to the *PBX1* t(1;19) breakpoints (P-1 and P-2). Sequence and QGRS scores for each motif we tested are listed in **Table 1**. Importantly, the sequence “T-5′ ” is 20 bases 5′ and “T-3′” is just 2 bases 3′ of a t(1;19) breakpoint cluster. T-3′(2) is a second G4 motif located ∼1200 bp from the major break site cluster. P-1 and P-2 are 70 and 750 bp from a t(1;19) break site cluster, respectively. However, unlike the breakpoints in the *TCF3* locus, the breakpoints in intron 1 of *PBX1,* involved with TCF3–PBX1 fusions, are more broadly distributed (Wiemels et al., 2002), suggesting that the G4 sequence motifs residing within the *PBX1* locus may not instigate translocations with *TCF3* to same degree as the *TCF3* G4 sequences.

**Table 1:** *TCF3* and *PBX1* G4 sequences.

Name	Sequence	QGRS score
T-5′-G4	CCA**GGGG**ACACT**GGG**TGATGTCT**GGGG**ACATCTACAGTTGTCA**GGG**CTGA**GGGG**AGC	62
T-3′-G4	AGA**GGG**AGAGA**GGG**AA**GGGGGG**A**GGG**C**GGGG**CA**GGG**CAG	72
T-3′(2)-G4	A**GGG**AGT**GGGG**ACGTGAAT**GGGG**TGCGA**GGGG**C**GGGG**TG	101
T-lg-G4	A**GGGGG**TGAGGC**GGG**AA**GGGG**ACAGCAGAACTCAC**GGGG**T	61
P-1-G4	GGT**GGGGG**CAGGTT**GGG**A**GGGG**AGGA**GGG**CAGATCTACA**GGG**A**GGG**TGG	70
P-2-G4	GCT**GGGG**T**GGGG**A**GGG**AAGAGATGA**GGGGG**A**GGG**AGA	66

*Names given for each sequence are in the far left column. Guanines that are predicted to be involved in G4 formation are bolded. QGRS scores are listed in the right column. The T-lg G4 sequence is not associated with t(1;19) break sites, it is a repeat motif located at the CNV site diagramed in **Figure 1***.

**FIGURE 2:**
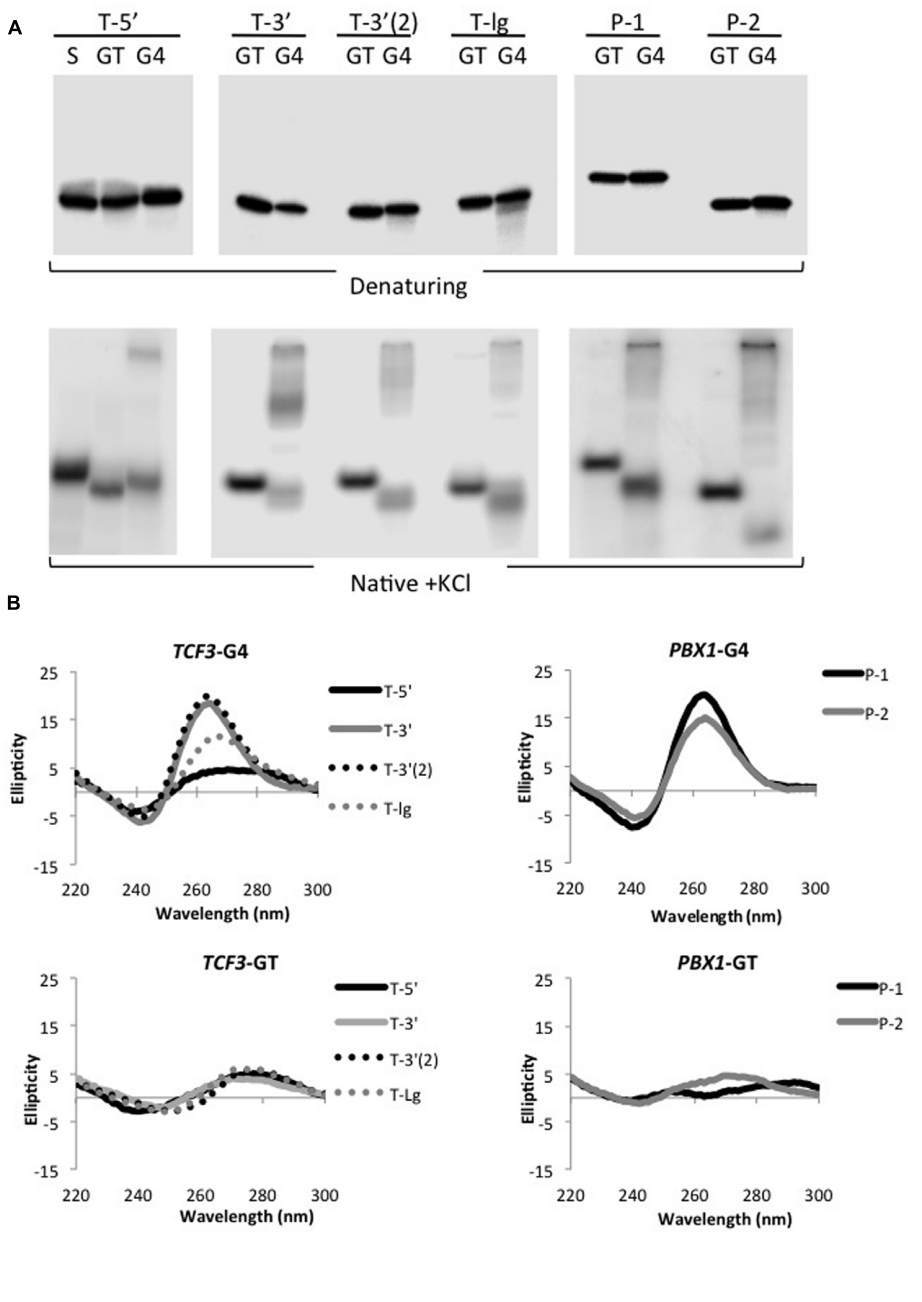
**Sequences from *TCF3* and *PBX1* adopt G4 conformations in solution. (A)** Phosphorimages showing 5′ end labeled guanine-rich oligonucleotides (G4) and corresponding thymine substituted controls (GT) resolved by PAGE. T-5′ also includes an additional scrambled control (S), where nucleotides were reordered to remove the potential for stable hairpin folding. Migration of each radiolabeled oligonucleotide upon denaturing PAGE (top) and native PAGE (bottom) is shown. **(B)** CD spectra for *TCF3* (top left) and *PBX1* (top right) G4 motifs. CD spectra are shown for thymine-substituted (GT) controls for *TCF3* (bottom left) and *PBX1* motifs (bottom right).

Each single-stranded guanine-rich motif (termed “G4”) was synthesized along with a companion control in which tandem guanine repeats of three or more were disrupted by substituting thymine, thereby greatly reducing the potential for G4 formation (termed “GT”; Supplementary Table S1). GT control and G4 motif oligonucleotides co-migrated on denaturing PAGE (**Figure 2A**, top), as expected. G4 DNA is stabilized by K^+^ or Na^+^ ions (Williamson et al., 1989; Sen and Gilbert, 1990). In the presence of 100 mM KCl, all of the *TCF3* and *PBX1* G4 oligonucleotides migrated as larger and smaller species compared to the GT interrupted control, which migrated as a single product in native PAGE (**Figure 2A**, bottom). The slow migrating species are consistent with multi-molecular structures, and species migrating faster than the GT control are consistent with mono-molecular, or self-pairing, conformations. GT control oligonucleotides retained identical mobility patterns in Native PAGE experiments independent of the presence of KCl (not shown), as expected. T-5′ retains some self-complementarity even when the guanine repeats were disrupted by thymine (GT control), likely explaining the faster mobility pattern observed in Native PAGE. Consistent with a mono-molecular structural conformation, scrambling the T-5′ sequence (T-5′-S) reduced its mobility compared to the companion GT and G4 samples (**Figure 2A**, bottom left). Together, we conclude that the guanine-rich sequences derived from *TCF3* and *PBX1* break site regions adopt alternative DNA conformations in the presence of K^+^. This is consistent with G4 DNA.

We further tested the ability of each *TCF3* and *PBX1* sequence motif to adopt G4 DNA structures in solution using circular dichroism (CD), comparing spectra of those oligonucleotides with controls that cannot adopt stable G4 conformations. CD measures the differential absorption of circularly polarized light by chiral molecules (called ellipticity). It is best suited for identifying the presence of structures, like G4, but not sensitive enough for atomic-level resolution. G4 DNA structures produce characteristic CD spectra, with positive peaks at either 264 or 295 nm and negative dips at 240 or 265 nm, respectively, depending on the type of quadruplex (Balagurumoorthy et al., 1992; Ðapić et al., 2003; Kypr et al., 2009; Vorlíčková et al., 2012). The CD spectra for all *TCF3* motifs tested showed molar ellipticities that peak at ∼264 nm and a dip at ∼240 nm (**Figure 2B**), consistent with G4 DNA. Interestingly, T-5′ shows a shallow and broadened peak that extends beyond 280 nm (**Figure 2B**, top left), probably reflecting the presence of non-G4, or B-form variant, structures (Kypr et al., 2009), which is consistent with Native PAGE analysis for this oligonucleotide (**Figure 2A**). CD analysis of T-5′ at a twofold higher concentration resulted in larger 260 nm maximum and 240 nm minimum peaks (Supplementary Figure S1), suggesting that at the lower concentration B-form conformations (i.e., self pairing) either reduces the potential for or competes with G4 DNA structure formation. Either way, the T-5′ sequence appears to adopt multiple DNA conformations that deviate from standard duplex. *PBX1* sequences P-1 and P-2 both showed CD spectra comparable to *TCF3* G4 DNA (**Figure 2B**, top right). As expected, interruption of the tandem guanine repeats by thymine substitution (Supplementary Table S1) eliminated the characteristic CD spectra for G4 (**Figure 2B**, bottom). We conclude that the sequences surrounding major break site regions in *TCF3* and *PBX1* adopt G4 DNA conformations in solution.

### *TCF3* and *PBX1* G4 Structures Block DNA Synthesis *In Vitro*

**FIGURE 3:**
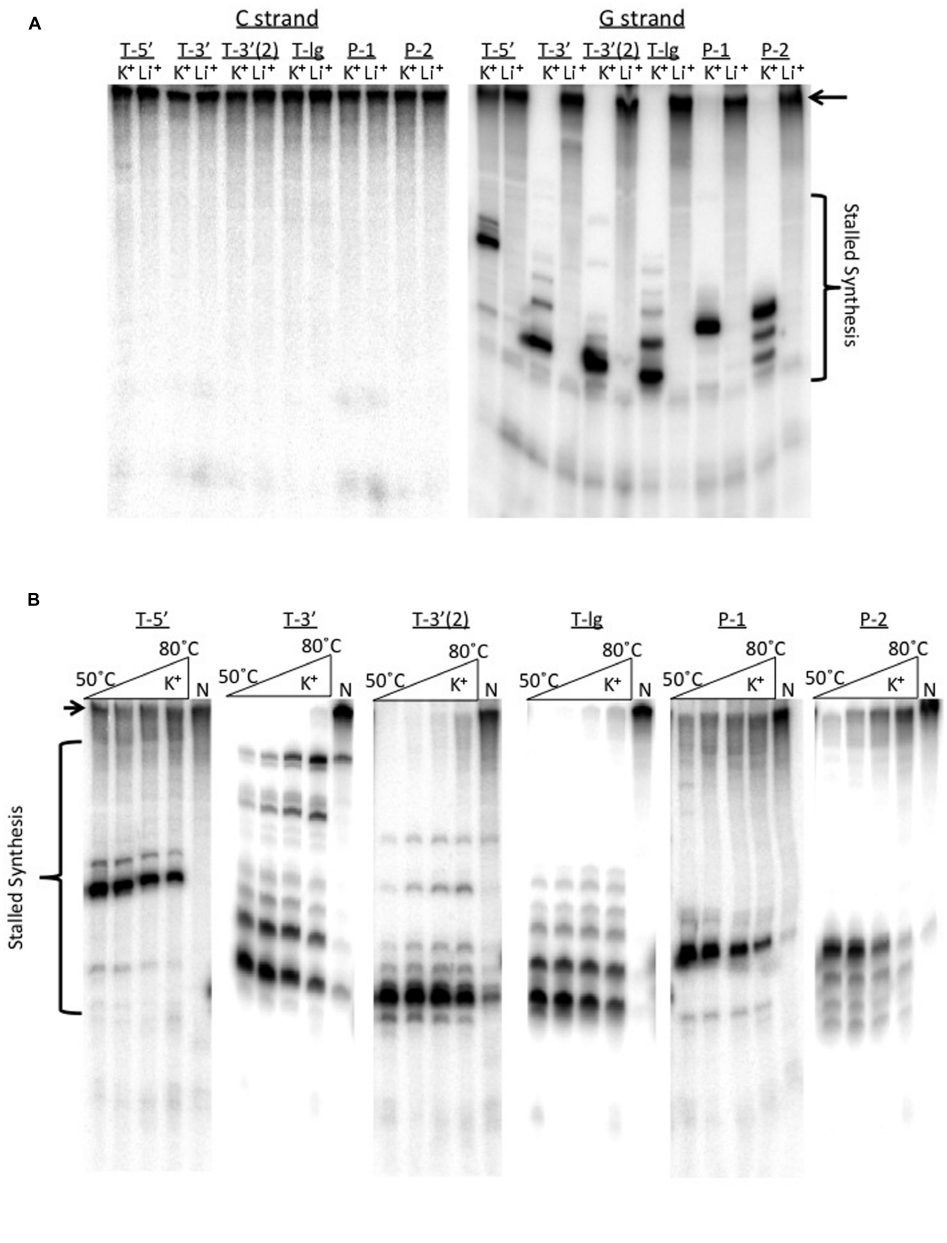
**Guanine-rich templates from *TCF3* and *PBX1* block DNA synthesis *in vitro*. (A)** Klenow polymerase extension assays using templates from the cytosine-rich strand (C-strand, left) or guanine-rich strand (G-strand, right) for each G4 sequence motif, resolved by denaturing PAGE. Reactions were performed in either KCl (K^+^) or LiCl (Li^+^). Shown are bands for stalled DNA synthesis (bracket) and full-length extension product (black arrow). G4 sequences begin at the bottom of the brackets. T-3′ differs from the motif listed in **Table 1**, it includes 472 bp of surrounding genomic sequence (Supplementary Figure S2). **(B)** Primer extension reactions used Taq polymerase across a temperature range (50–80∘C) in either KCl (K^+^) or (NH_4_)_2_SO_4_ (N) salt conditions on guanine-rich templates from *TCF3* and *PBX1*. Bands corresponding to stalled DNA synthesis (bracket) or full-length extension (black arrow) are shown.

The precise mechanisms by which formation of G4 DNA structures induce genome instability are not defined. Hotransiently denatured have an opportunity to interact, or self pair, to form structures that interfere with DNA metabolism (Zhao et al., 2010; Lopes et al., 2011; van Kregten and Tijsterman, 2014). Presumably, highly stable non-duplex DNA structures are more likely to interfere with DNA transactions compared with those that are less stable. To test that model for *TCF3* G4 sequence motifs we next employed primer extension assays using genomic sequences surrounding the major break sites. This assay has been well described for characterizing G4 formation, showing that K^+^ ions support guanine-dependent G4 formation and polymerase pausing, while Li^+^ and NH_4_^+^ ions do not (Weitzmann et al., 1996; Sun and Hurley, 2010; Ehrat et al., 2012). Using that system, we expected to find K^+^-dependent polymerase pausing on templates containing *TCF3* and *PBX1* break site sequences. Templates for this assay contained the G4 motifs shown in **Table 1**, but also some additional genomic sequence (Supplementary Figure S2). We cloned each sequence into the plasmid pCR2.1 (Invitrogen, Carlsbad, CA, USA) in both orientations with respect to an F1 origin and the primer-binding site. Single-stranded phagemids were then isolated for each orientation (cytosine-rich or guanine-rich sequences) and used as single-stranded templates for primer extension reactions catalyzed by Klenow polymerase. Independent of specific template, polymerase extension reactions showed full-length product when the cytosine-rich strands (complements to the G4 motifs) were assayed, and synthesis was independent of the salt used, as expected (**Figure 3A**, left and Supplementary Figure S3). In contrast, extension reactions prematurely paused on the guanine-rich templates when K^+^, but not Li^+^, was present (**Figure 3A**, right). Although fully consistent with G4 DNA, we further tested the dependence of stalling on guanine by using thymine substitution mutagenesis to disrupt the repeats in the T-5′, P-1, and P-2 templates (Supplementary Figure S2). Full extension was restored when the G4 motifs were disrupted (Supplementary Figure S4), essentially matching results for the C-rich complements (**Figure 3A**), and demonstrating that the guanine repeats are needed for the polymerase pausing. Therefore, this stalling of synthesis argues that G4 structures can form from the *TCF3* and *PBX1* break site sequences.

We next asked if the G4 structures formed within *TCF3* and *PBX1* sequences are thermally stable, and thereby capable of adopting difficult to resolve structural conformations in the cell. Taq polymerase, used in standard PCR, has optimal activity around 75∘C (Lawyer et al., 1993). One would predict that at elevated temperatures, beyond 37∘C, any polymerase pausing would reflect the presence of highly stable template blockades. Based on that logic, we replicated the primer extension experiments described above with Taq polymerase at ranging temperatures in reactions containing either K^+^ or NH_4_^+^ salt. Resolution of the Taq extension products by denaturing PAGE revealed polymerase-stalling patterns similar to that of Klenow (**Figure 3B**). Importantly, bands corresponding to stalled synthesis on the guanine-rich templates were only marginally altered when reaction temperatures reached 80∘C, suggesting that the replication blockades formed within the *TCF3* and *PBX1* templates are thermally stable (**Figure 3B**). Full extension was observed when NH_4_^+^ was substituted for K^+^ (**Figure 3B**) or when the C-rich strand served as the template (Supplementary Figure S3), as expected. We conclude that the G-rich sequence motifs located proximal to the *TCF3* and *PBX1* t(1;19) translocation sites (**Figure 1**) and to the *TCF3* CNV break sites (**Figure 1A**) all form G4 structures capable of interfering with DNA synthesis *in vitro*.

### *TCF3* Break Site G4 Motifs and Instability

**FIGURE 4:**
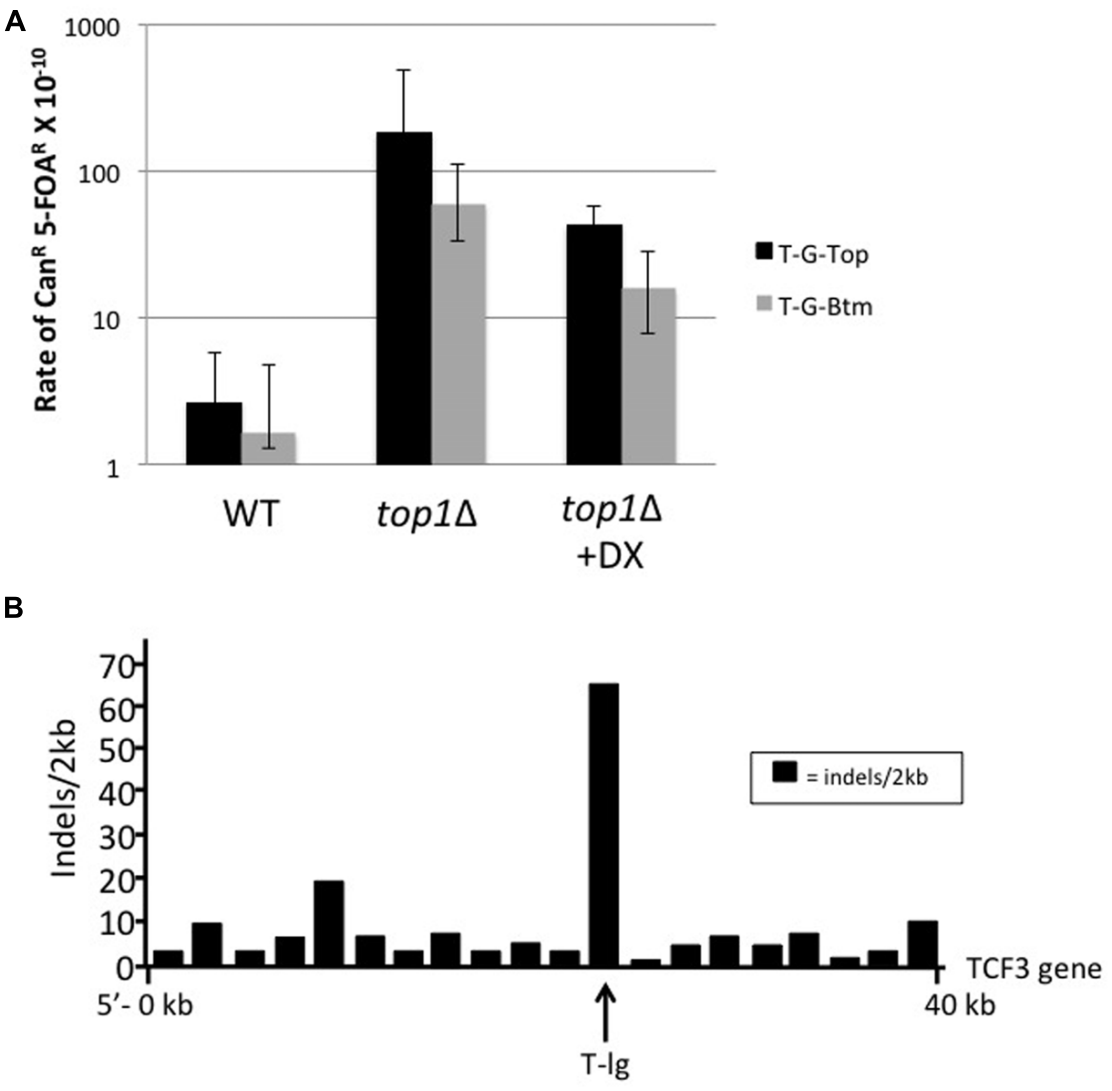
***TCF3* G4 motifs promote genetic instability in yeast. (A)** Rate of gross chromosomal rearrangements for the t(1;19) major break site sequence (T-3′-G4 motif) in the *pTET-lys2 T-G-Top* (solid black bar) or *T-G-Btm* (gray bar) for WT or *top1* deletion yeast strains. Where indicated (+DX), transcription was repressed by addition of doxycycline to 2 mg/l. Error bars indicate 95% confidence intervals. **(B)** *TCF3* intron with high CNV (T-lg) also shows high levels of small insertions and deletions (<100 bp). Insertion and deletions (indels) identified in *TCF3* using 2 kb sequence windows are mapped (indels/2 kb) according to genetic position.

Although our *in vitro* results suggests that sequences proximal to *TCF3* breaks adopt G4 DNA, it does not necessarily connect structure formation with site-specific instability in the cell. Therefore, we next asked if the G4 motifs surrounding the *TCF3* t(1;19) break site promote instability in yeast using a previously described genetic assay. In these systems, a model G4-forming sequence from the murine Sμ Ig switch region was shown to enhance ectopic recombination (Kim and Jinks-Robertson, 2011) and gross chromosome rearrangements (GCRs; Yadav et al., 2014). The genome instability occurring at the Sμ Ig switch region was influenced by conditions that impact G4 structure formation, such as high transcription rate, orientation of the sequence with respect to the promoter, and disruption of structure metabolizing enzymes like Sgs1, a G4 specific helicase (Huber et al., 2002), or Top1 topoisomerase (Yadav et al., 2014). In order to test whether G4 motifs identified at the *TCF3* translocation breakpoints can also induce genome instability, we modified the GCR assay, which selects for the simultaneous loss of the *CAN1* and *URA3* genes located telomeric to a reporter cassette that is integrated on Chromosome V. This cassette consists of *LYS2* gene transcribed from the tetracycline-repressible promoter *pTET*. A 472 bp sequence (T-3′-G4 Supplementary Figure S2) surrounding the *TCF3* t(1;19) G4 motif was introduced into this cassette so that the G-rich strand is positioned on either the non-transcribed (T-G-Top) or transcribed (T-G-Bottom) strand with respect to the *pTET* promoter. In wild type backgrounds under high transcription conditions (WT), the rates of GCR for T-G-Top and T-G-Bottom did not differ significantly (**Figure 4A**). GCR rates increased significantly for both T-G-Top and T-G-Bottom by 69- and 36-fold, respectively, in Top1-deficient yeast strains. This is similar to the G4-associated genetic instability observed for the model G4 sequence, Ig Sμ (Yadav et al., 2014). When the transcription from *pTET* was repressed by addition of tetracycline analog doxycycline (+DX), the rates of GCR for T-G-Top and T-G-Btm were both reduced by ∼4-fold compared to the high transcription conditions (**Figure 4A**). The difference in instability between T-G-Top and T-G-Bottom in both transcription conditions ranged from ∼2.7 to 3-fold. Therefore, in this assay T-G-Top, which is in a G4 favorable orientation, promoted the formation of DNA breaks leading to chromosomal rearrangements. However, transcriptional orientation had less of an impact on instability compared to the model Sμ G4 sequence, with about a 28-fold difference between the two Sμ transcriptional orientations (Yadav et al., 2014) compared to threefold difference for *TCF3* under high transcription conditions (**Figure 4A**). This probably reflects the difference in the density of G-runs and the relative sizes of the *TCF3* and Sμ sequences used in the assay. Regardless, the t(1;19) G4 break site sequence from *TCF3* displayed co-transcriptional genetic instability, fully consistent with the model that G4 DNA formation promotes *TCF3* instability.

The second site of instability in *TCF3* (T-lg) is associated with CNVs (**Figure 1**), and those sequences readily adopted G4 structures *in vitro* (**Figures 2** and **3**). We next asked if this site in *TCF3* contains signatures for G4-mediated instability by using the existing genome variation databases. T-lg is extensively repetitive (with 106 GGG repeats), so it is reasonable to predict that the CNVs mapping to this region are associated with DNA breaks related to the G4 motifs and G4 structure formation. If so, we would expect to find DNA break signatures proximal to the tandem guanine repeats. Therefore, we examined the entire *TCF3* gene using the human database for short genetic variation (dbSNP; Sherry et al., 2001) available on Ensembl77 (Kersey et al., 2014), looking specifically to map the positions of short (<100 bp) insertions and deletions. At the CNV site (T-lg), there are approximately 8-fold more insertions and deletions (indels) compared to the rest of *TCF3* (**Figure 4B**). The locations of the sequence variations are not random, with 93% of indels directly next to or inside of a tandem guanine repeat (Supplementary Figure S5). Fine mapping of deletion positions shows that all directly flank or reside within the same guanine repeat sequence, which is part of a sequence motif repeated 32 times in the T-lg sequence (Supplementary Figure S6). Insertion mutations were also distributed within two nucleotides of the tandem guanine repeats, but there are fewer insertions overall compared to deletions (Supplementary Figure S6). Although it is not immediately clear what the pattern or type of sequence variation indicates with regard to the mechanism(s) of instability, the loss, and gain of sequence coincides with repetitive guanines.

We favor a model whereby guanine-rich DNA in *TCF3* adopts G4 DNA structures, and failure to resolve those structures promotes DNA breaks. *TCF3* instability due to G4 formation is manifested in the available genome databases as copy number variations and translocation events. This is significant because it is currently unclear why the *TCF3* gene is unstable. An alternate possibility to consider is that the guanine repeats in *TCF3* alone are unstable, or that instability is otherwise independent of G4 structure formation. When long guanine-rich sequences are positioned on the non-transcribed strand the cytosine rich complement can participate in RNA–DNA hybridizations during transcription to form structures called R-loops, which promote instability (Hamperl and Cimprich, 2014). Indeed, recent results suggest that R-loops are cleaved by factors required for transcription-coupled nucleotide excision repair, providing a potential mechanism for instability at guanine-rich DNA (Sollier et al., 2014). G4 can form on the unpaired strand, and those structures were observed in electron microscopy studies of transcribed switch regions (Duquette et al., 2004), although there is also evidence that R-loop stability may not depend upon G4 formation (Roy et al., 2008). We did not directly test the capacity of various *TCF3* sequences to form R-loop structures in this work. However, we did observe that instability was influenced by transcription and the transcriptional orientation of T-3′ had a modest affect (∼3-fold) on gross chromosomal loss in yeast (**Figure 4A**). Therefore, we cannot exclude the possibility that R-loop formation itself contributes to *TCF3* instability at some level.

Our findings add to a growing list of oncogenic rearrangements that have been connected with G4 DNA structures. G4-forming sequences are found in the *BCL-2* and *HOX11* genes near oncogenic translocation sites (Nambiar et al., 2011, 2013). And a region of the unstable *c-MYC* oncogene also adopts G4 *in vitro* (Simonsson et al., 1998). G4 formation may be common among oncogenes participating in rearrangements because previous analyses of sequences at translocation breakpoint regions revealed G4 sequences in multiple genes associated with lymphoid cancers, including *TCF3* (Katapadi et al., 2012). Results presented here demonstrate that guanine repeat sequences at two different sites of instability in the *TCF3* gene are able to fold into stable G4 structures *in vitro*, suggesting a role for this structure in promoting the genetic instability of *TCF3*. Our results provide one molecular rationale for the apparent instability of *TCF3*. Mutagenesis and recombination at *TCF3* may not be due to the sequences found at the break sites *per se*, but rather the capacity of these sequences to transform into G4 DNA conformations.

## Materials and Methods

### Sequence Analysis

The *TCF3*/*PBX1* t(1:19) breakpoint sequences from the Lieber database (Wiemels et al., 2002; Tsai et al., 2008) and the TICdb (Novo et al., 2007) were mapped onto *TCF3* and *PBX1* genome sequences. CNVs for *TCF3* were downloaded from the database of genomic structural variation (dbVAR) on NCBI. org (Lappalainen et al., 2013). All CNVs’ breakpoints (>99 bp) were mapped to *TCF3*’s genomic location and confirmed using Ensembl release 77 (Kersey et al., 2014). For the identification of G4 sequence motifs, *TCF3* and *PBX1* sequences were analyzed using QGRS mapper (Kikin et al., 2006) with the following filters: a maximum loop length of 45 nucleotides, minimum G group of 3, and a loop size 0–36 nucleotides. The output of that analysis was mapped to *TCF3* and *PBX1* genes. G4 sequence motifs with a QGRS score of at least 42 were presented as the number of independent G4 sequences identified in 2 kb non-overlapping windows. Guanine repeat motifs used in structure analysis were selected based on their proximity to the t(1;19) breakpoint clusters in *TCF3* and *PBX1* (T-5′, T-3′, T-3′(2), P-1, and P-2), and to breaks associated with copy number variations identified for *TCF3* (T-lg). The location of all dbSNP database insertions and deletions (Sherry et al., 2001) were mapped to *TCF3* using Enseml release 77 (Kersey et al., 2014) and density was calculated by number of insertion or deletion events per 2 kb.

### G4 Folding and PAGE Analysis

G4 oligonucleotides for native PAGE and CD analysis were synthesized and PAGE purified by Operon (Huntsville, AL, USA). Sequences are shown in **Table 1** and Supplementary Table S1. For Native PAGE, oligonucleotides were 5′ end labeled using T4 PNK (New England Biolabs, Ipswich, MA, USA) and [γ-P^32^]ATP (MP Biomedicals, Solon, OH, USA) at 37∘C for 30 min. Unincorporated label was removed by Illustra Microspin G-25 spin chromatography (GE healthcare, Pittsburgh, PA, USA). G4 structures were formed in reactions containing 100 mM KCl in Tris-EDTA buffer by incubating in a small >90∘C water bath that was allowed to cool slowly to room temperature. Samples were then incubated an additional hour at 37∘C. Native PAGE experiments used 16% polyacrylamide (37:1) containing 0.5 X TBE with 100 mM KCl in the gel and run buffer. Oligonucleotides were resolved by electrophoresis at 100 V at room temperature for 6 h. Denaturing PAGE of radiolabeled oligonucleotides used 16% polyacrylamide (19:1) gels made with 7 M urea and 0.5X TBE. Prior to loading, samples were denatured in 90% formamide and heated to 90∘C for 20 min. DNA was resolved by electrophoresis at 400 V for 1.5 h. Images were captured by phosphorimaging using a Molecular Dynamics Storm 840 phosphorimager (Amersham/GE). Each PAGE analysis was repeated at least three times and representative images are shown.

### Circular Dichroism

Circular dichroism analysis was performed using an Aviv model 215 CD spectrometer at 37∘C. Spectra were taken in 1 cm path quartz cells containing 12 μM G4 or GT oligonucleotide in 10 mM Tris-HCl, pH 7.6, 1 mM EDTA, and 100 mM KCl. The molar ellipticity was measured from 220 to 300 nm and recorded for three scans in 1 nm increments at a 1 s averaging time.

### Primer Extension Assays

Phagemids for extension assays were obtained by reconstituting the genomic sequence via overlapping PCR using semi-complimentary primers in a standard PCR reaction. PCR products were gel purified and TOPO cloned (Invitrogen) into pCR2.1 in both orientations and verified by sequencing. Templates for extension assays included the sequences shown in **Table 1** and were the following sizes; T-5′ (161 bp), T-3′ (472 bp), T-3′(2) (168 bp), T-lg (124 bp), P-1 (95 bp), P-2 (92 bp; Supplementary Figure S2). Closed circular single stranded DNA was obtained using M13K07 helper phage (NEB) according to the manufacturer’s instructions.

Polymerase extension assays were performed essentially as described (Ehrat et al., 2012) and based on previously described assays (Weitzmann et al., 1996; Sun and Hurley, 2010). Single-stranded phagemid templates were primed with a ^32^P 5′ end labeled M13 forward primer, which was extended with Klenow or Taq polymerase (NEB). In Klenow reactions, KCl or LiCl was added to a final concentration of 25 mM. Klenow extension reactions took place at 37∘C for 8 min on single stranded template primed with 5′ end-labeled M13 forward (-20) primer. Taq reactions used identical conditions, except that temperatures ranged from 50 to 80∘C for 9 min in buffers containing either (NH_4_)_2_SO_4_ or KCl salt. Extension reactions were halted by the addition of an equal volume of 90% formamide and 1 mM EDTA followed by heating to 90∘C for 20 min. Products of polymerase extension were resolved by 8% denaturing PAGE (19:1) with 7 M urea and 0.5X TBE, at 700 V at room temperature. Gels were then dried and images were captured by phosphorimaging with a Molecular Dynamics Storm 840 phosphorimager (Amersham/GE). Primer extension reactions were repeated three times and representative images are shown.

### Gross Chromosomal Rearrangements Assay

The plasmids containing *lys2 T-G-Top* and *lys2 T-G-Btm* cassettes were constructed by inserting the 472 bp T-3′ sequence (Human genome 1:1618084-1618556) into the BglII site located +390 nt position within the *S. cerevisiae LYS2* gene in two different orientations relative to transcription start site. Using the standard two-step allele replacement protocol, these constructs were used to replace the wild type *LYS2* gene located proximal to *CAN1* on the left arm of the chromosome V. The rates of GCR occurring at the chromosome V were determined according to the previously described procedure (Yadav et al., 2014). Briefly, individual colonies were inoculated into the rich media (1% yeast extract, 2% peptone, and 2% glucose – YEPD) and cultured to saturation at 30∘C. Appropriate dilution of the cultures was plated either on the YEPD media for determination of total cell numbers or on the selective media (synthetic complete media supplemented with canavanine (60 mg/l) and 5-Fluoroorotic acid (1 g/l) for determination of the cells that lost *CAN1* and *URA3* genes. For each strain, 24–32 cultures were used to calculate rate and 95% confidence levels either by Lea-Coulson method of median (*top1Δ,* high transcription; Spell and Jinks-Robertson, 2004) or by P_0_ method (*wt*, high transcription, *top1Δ* low transcription; Foster, 2006).

## Author Contributions

JW, experimental design, experimentation, and manuscript preparation. AB, experimental design and experimentation. SF, experimental design, experimentation, and manuscript preparation. EL, research design and manuscript preparation. All authors read and approved the final manuscript.

## Conflict of Interest Statement

The authors declare that the research was conducted in the absence of any commercial or financial relationships that could be construed as a potential conflict of interest.

## Abbreviations

ALL: acute lymphoblastic leukemia
CD: circular dichroism
CNV: copy number variant
G4: guanine quadruplex
PBX1: Pre-B cell leukemia homeoboX 1 gene
TCF3: transcription factor 3 (E2A)
