# Supplementary material for: Sites of instability in the human TCF3 (E2A) gene adopt G-quadruplex DNA structures in vitro
Source: Front Genet. 2015 May 11;6:177. doi: 10.3389/fgene.2015.00177 (PMC4426816; doi:10.3389/fgene.2015.00177)
Supplement: Supplementary file 1 [file Image_1.PDF]

Supplementary Material

Sites of instability in the human *TCF3* (*E2A*) gene adopt G-quadruplex DNA structures in vitro

Jonathan D. Williams[1], Sara Fleetwood[1], Alexandra Berroyer[1], Nayun Kim[2], and Erik D. Larson[1*]

[1] School of Biological Sciences, Illinois State University, Normal, IL, USA

[2] Department of Microbiology and Molecular Genetics, University of Texas Health Science Center at Houston, Houston, TX, USA

* Correspondence: Erik Larson, Illinois State University, School of Biological Sciences, Campus Box 4120, Normal, IL, 61790-4120, USA.
elarson@ilstu.edu

Name	Sequence	QGRS score
T-5'-S	GACTGATCAGGTGAGCGGCGTAGTAGCTGGTGCTAAGGCGTGAGGAGGTGGCATCCC	0
T-5'-GT	CCAGTTGACACT**GGG**TGATGTCTGTTGACATCTACAGTTGTCAGTGCTGA**GGGG**AGC	0
T-5'-G4	CCA**GGGG**ACACT**GGG**TGATGTCT**GGGG**ACATCTACAGTTGTCA**GGG**CTGA**GGGG**AGC	62
T-3'-GT	AGAGTGAGAGAGTGAAGGTGTGAGTGCGTGGCAGTGCAG	0
T-3'-G4	AGA**GGG**AGAGA**GGG**AA**GGGGGG**A**GGG**C**GGGG**CA**GGG**CAG	72
T-lg-GT	A**GGTGG**TGAGGC**GTG**AA**GTGG**ACAGCAGAACTCAC**GTGG**T	0
T-lg-G4	A**GGGGG**TGAGGC**GGG**AA**GGGG**ACAGCAGAACTCAC**GGGG**T	61
T-3'(2)-GT	AGTGAGTGTTGACGTGAATGTTGTGCGAGTTTCGTTGTG	0
T-3'(2)-G4	A**GGG**AGT**GGGG**ACGTGAAT**GGGG**TGCGA**GGGG**C**GGGG**TG	101
P-1-GT	GGTGTTGGCAGGTTGTGAGTTGAGGAGTGCAGATCTACAGTGA**GGG**TGG	0
P-1-G4	GGT**GGGGG**CAGGTT**GGG**A**GGGG**AGGA**GGG**CAGATCTACA**GGG**A**GGG**TGG	70
P-2-GT	GCTGTTGTGTTGAGTGAAGAGATGAGTTGGA**GGG**AGA	0
P-2-G4	GCT**GGGG**T**GGGG**A**GGG**AAGAGATGA**GGGGG**A**GGG**AGA	66

Supplementary Table 1. Oligonucleotide sequences. *TCF3* and *PBX1* genomic sequences (G4) and companion controls (S and GT) used in G4 structure formation assays (center), QGRS scores (right). T-5’-scambled (S) is an additional control sequence that has a rearranged sequence to prevent hairpin or G4 structure formation.

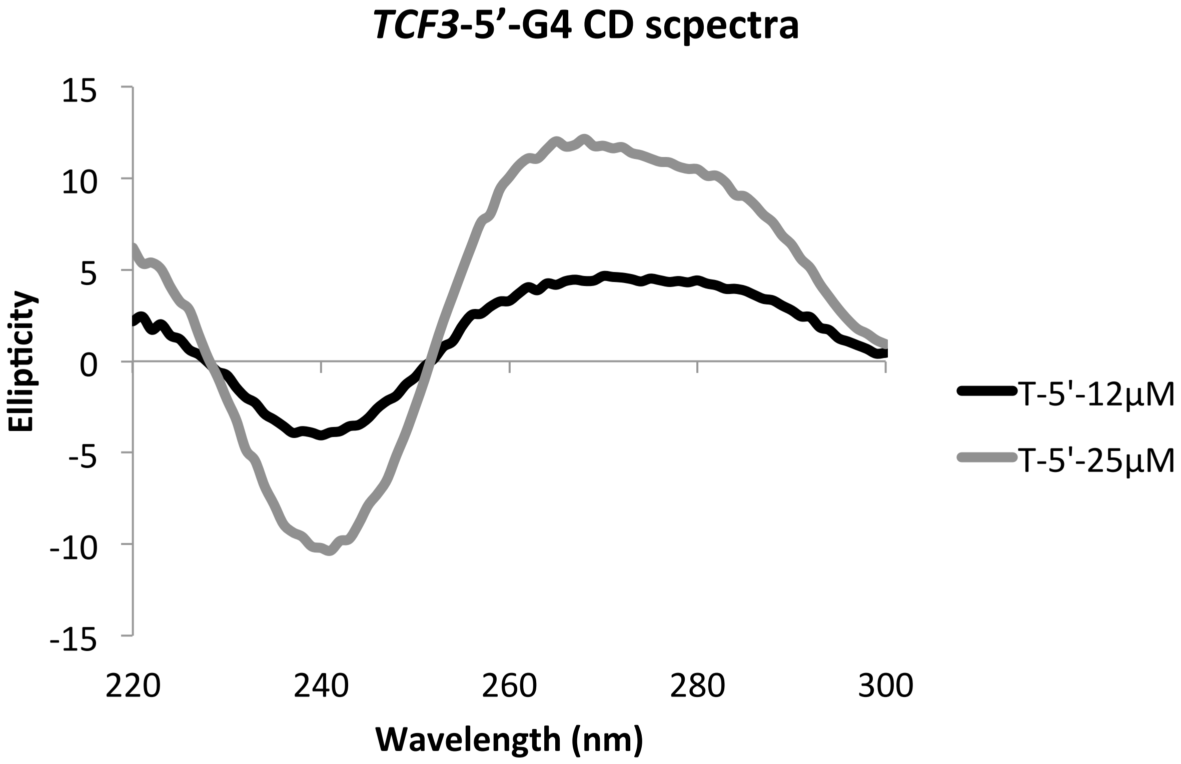

Supplementary Figure 1. CD spectra for T-5’-G4 CD spectra for T-5’-G4 are shown at 12 µM and 25 µM oligonucleotide concentrations.

Sequences for primer extension assays

T-5′-G4
CCA**GGGG**ACACT**GGG**TGATGTCT**GGGG**ACATCTACAGTTGTCA**GGG**CTGA**GGGG**AGCTCCTGGCATGGAGT**GGG**T**GGGG**
GCCA**GGG**ATGCTGCTCAGCACCCTGCAGTGCCCAGGACGGCCCCACCCCAGAGAA**GGG**TCCGGACCCACAAA**GGG**C

T-5′-GT
CCA**GGGG**ACACT**GGG**TGATGTCT**GGGG**ACATCTACAGTTGTCAGTGCTGAGGTGAGCTCCTGGCATGGAGTGTGTGGTG
GCCA**GGG**ATGCTGCTCAGCACCCTGCAGTGCCCAGGACGGCCCCACCCCAGAGAA**GGG**TCCGGACCCACAAA**GGG**C

T-3′-G4
GGGGTTGGAGCAGAGTGAGGAGA**GGG**AGAGA**GGG**AA**GGGGGG**A**GGG**C**GGGG**CA**GGG**CAGGTCATGC**GGGG**CCTTGT**GGG**
CTGC**GGGG**AGGACTT**GGG**ATTTGGCCATGAGAAAGGTGGCAGCCGTGGA**GGG**CTGAGGA**GGG**AT**GGG**ACCTGACCCAGG
TGCTCACAGATACCCTCTGGTGGCTGCTTGGAGGACAGACTATGAGTGGCACC**GGG**AGACCAAGGCAGAGGCCACA**GGG**
CTGGTCCA**GGGG**CCTTGGA**GGGG**TGAGCAGT**GGG**T**GGG**CCCTGNATCTCCTGAAGGC**GGGG**CCCACAGGATTTGTGATG
GACAGGACTTGA**GGG**TGAGAGAAGGCA**GGG**TGGCTCT**GGG**ATTTCTGGCCCGAGCTGT**GGG**TGACACT**GGGG**CT**GGG**AA
CTCCGTAAGGAA**GGGG**ACAAAGGAAAAGGTT**GGGG**ACAAATCTGGACCT**GGG**CCT**GGGG**ATTGTTAA**GGG**C

T-3′(2)-G4
CTGTC**GGGG**AA**GGG**T**GGGG**T**GGGG**C**GGGG**CAGGCACTCACCAGGCCGGAGACCCCCGTCGTAGCT**GGG**CGATAAGGCAC
C**GGGGG**CTCCTGCTCGAGGCCACTGTGACGTTCCTGGAA**GGG**AGT**GGGG**ACGTGAAT**GGGG**TGCGA**GGGG**C**GGGG**TGTA
A**GGG**

T-lg-G4
T**GGGG**ATGAGGC**GGG**AA**GGGG**ACAGCAGAGCTCACA**GGGGG**TGAGGC**GGG**AA**GGGG**ACAGCAGAACTCAC**GGGG**TGAGG
C**GGG**AA**GGGG**ACAGCAGAGCTCACA**GGGGG**TGAGGCGGAAA**GGGG**

PBX1-1-G4
CCACAGGT**GGGGG**CAGGTT**GGG**A**GGGG**AGGA**GGG**CAGATCTACA**GGG**A**GGG**TGGTCTTCAGATTTGGACAACAGTCCAA
GGGCGCC

PBX1-1-GT
CCACAGGTGTTGGCAGGTTGTGAGTTGAGGAGTGCAGATCTACAGTTAGGGTGGTCTTCAGATTTGGACAACAGTCCAA
GGGCGCC

PBX1-2-G4
ATTTCAGAAAAAAGCTGCT**GGGG**T**GGGG**A**GGG**AAGAGATGA**GGGGG**A**GGG**AGAGAGCGCA**GGG**CACCCATCA**GGG**AAAA
GGGC

PBX1-2-GT
ATTTCAGAAAAAAGCTGCTGGTGTGGTGAGTGAAGAGATGAGTTGGAGTGAGAGAGCGCAGTGCACCCATCAGTGAAAA
GGGC

Supplementary Figure 2. Sequences for templates used in polymerase extension assays. Genome sequences PCR amplified or synthesized were cloned into pCR2.1, and include G4 motifs from *TCF3* or *PBX1* plus additional surrounding genomic sequence. Only the guanine-rich templates are shown, the complements are cytosine rich.

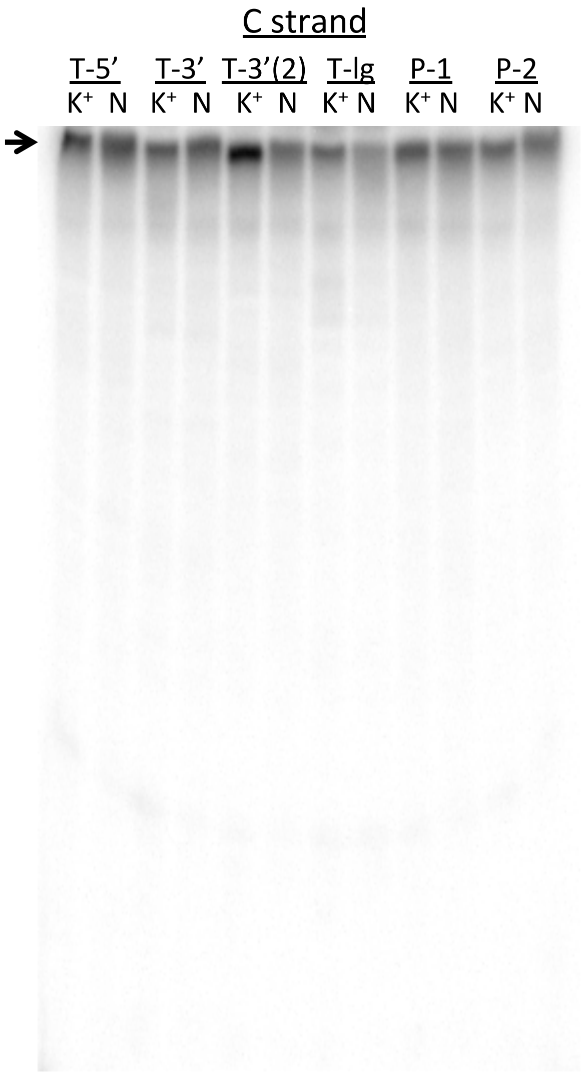

Supplementary Figure 3. Cytosine-rich templates from *TCF3* and *PBX1* do not stall Taq polymerase. Taq polymerase extension assay using templates from the complementary cytosine-rich strand (C-strand) of each G4 sequence motif was resolved by denaturing PAGE and full-length extension products (arrow) are shown. Either KCl (K^+), or $(NH_4)_2SO_4$ (N) salt conditions are used.

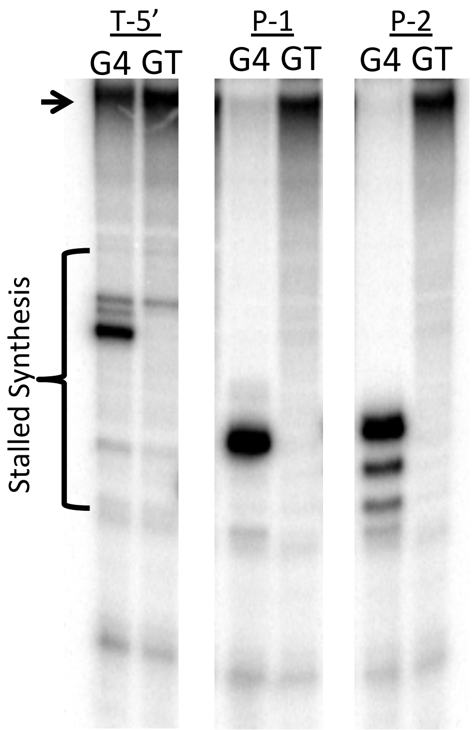

Supplementary Figure 4. Polymerase pausing *in vitro* is dependent on guanine repeats. Klenow polymerase extension assays using guanine-rich (G4), or guanine substituted (GT) templates for T-5’, P-1 and P-2 sequences. Full primer extension products (arrow) and polymerase pausing, or stalled synthesis (bracket) are shown on the left.

Supplementary Figure 5. Sequence location of T-lg insertions and deletions in respect to guanine runs. Ensembl77 was used to map location of insertions and deletions (indels) from the dbSNP database. Actual T-lg sequence shown with deletions and insertions identified denoted to the corresponding nucleotide.

GAGCATCTGCAGCCCACAGAAGCCCTGCCCAGGGGGTGGGGCGGAAAGGGGACAGCAGAGCTCACGGGGTGAG
GTGGGAAGGGGACAGCAGAGCTCACAGGGGGTGAGGCGGGAAGGGGACAGCAGAGCTCACAGGGGGTGAGG
CGGGAAGGGGACAGCAGAGCTCACAGGGGGTGAGGCGGGAAGGGGACAGCAGAGCTCACAGGGGGTGAGGC
GGGAAGGGGACAGCAGAGCTCACAGGGGATGAGGCGGGAAGGGGACAGCAGAGCTCACGGGGTGAGGCGGG
AAGGGGACAGCAGAGCTCACAGGGGGTGAGGCGGGAAGGGGACAGCAGAGCTCACAGGGGGTGAGGCGGGA
AGGGGACAGCAGAGCTCACGGGGGTGAGGCGGGAAGGGGACAGCAGAGCTCACAGGGGATGAGGCGGGAAG
GGGACAGCAGAACTCACGGGGTGAGGCGGGAAGGGGACAGCAGAGCTCACGGGGTGAGGCGGGAAGGGGAC
AGCAGAGCTCACAGGGGGTGAGGCGGGAAGGGGACAGCAGAGCTCACGGGGGTGAGGCGGGAAGGGGACAG
CAGAGCTCACGGGGTGAGGCGGGAAGGGGACAGCAGAGCTCACAGGGGATGAGGCGGGAAGGGGACAGCAGA
GCTCACAGGGGGTGAGGCGGGAAGGGGACAGCAGAACTCACGGGGTGAGGCGGGAAGGGGACAGCAGAGCTC
ACAGGGGGTGAGGCGGAAAGGGGACAGCAGAGCTCACAGGGGATGAGGCGGGAAGGGGACAGCAGAGCTCAC
AGGGGGTGAGGCGGGAAGGGGACAGCAGAGCTCACGGGGGTGAGGCGGGAAGGGGACAGCAGAGCTCACGG
GGGTGGGGCAGGAAGGGGACAGCAGAGCTCACAGGGGGTGAGGCGGGAAGGGGACAGCAGAACTCACGGGG
TGAGGCGGGAAGGGGACAGCAGAGCTCACAGGGGGTGAGGCGGAAAGGGGACAGCAGAGCTCACAGGGGATG
AGGCGGGAAGGGGACAGCAGAGCTCACAGGGGGTGAGGCGGGAAGGGGACAGCAGAGCTCACAGGGGATGA
GGCGGGAAGGGGACAGCAGAACTCACGGGGTGAGGCGGGAAGGGGACAGCAGAGCTCACGGGGTGAGGCGG
GAAGGGGACAGCAGAGCTCACGGGGTGAGGCGGGAAGGGGACAGCAGAACTCACGGGGTGAGGCGGGAAGG
GGACAGCAGAGCTCACGGGGTGAGGCGGGAAGGGGACAGCAGAGCTCACAGGGGGTGAGGCGGGAAGGGGA
CAGCAGAGCTCACGGGGGTGAGGCGGGAAGGGGACAGCAGAGCTCACGGGGTGAGGCGGGAAGGGGACAGCA
GAGCTCACGGGGTGAGGCGGGAAGGGGACAGCAGAGCTCACGGGGTGAGGCGGGAAGGGGAC

(arrow) =insertion
N = deleted once
N (single slash) = deleted twice
N (cross) =deleted three times
GGG = Guanines involved in G4

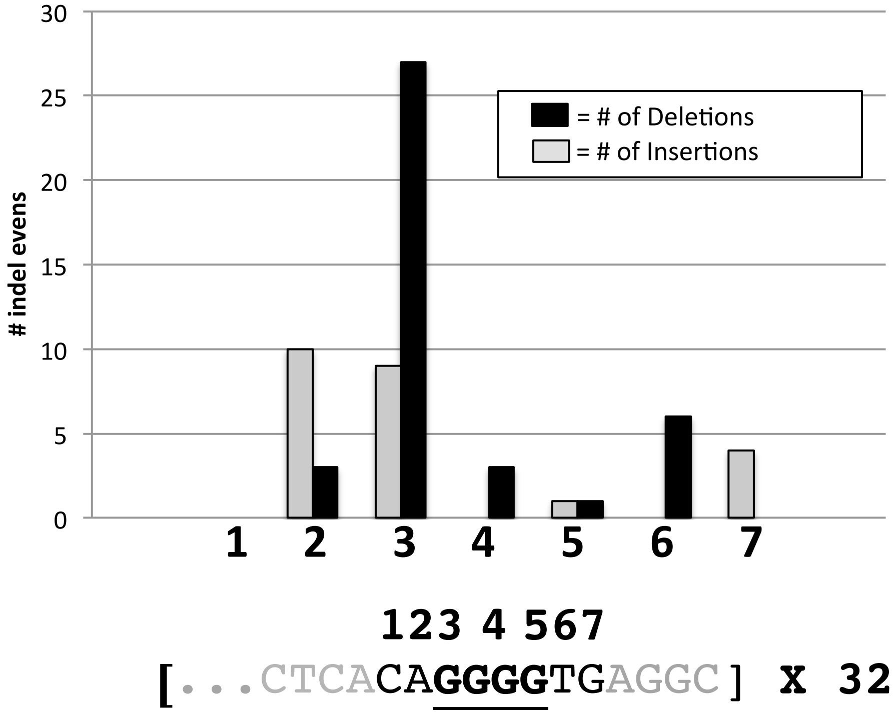

Location of indels compared to the repetitive sequence of T-lg

Supplementary Figure 6. Graphed location of T-lg insertions and deletions in respect to guanine runs.

Fine mapping of insertions and deletions with respect to the guanine repeat unit composing the T-lg intron. Numbers above the repeat motif correlate with numbers on the graph, depicting the location for each deletion (black bar) or insertion (grey bar) in the T-lg repeat motif.
